# Resveratrol distinctively modulates the inflammatory profiles of immune and endothelial cells

**DOI:** 10.1186/s12906-017-1823-z

**Published:** 2017-06-13

**Authors:** Joseph Schwager, Nathalie Richard, Franziska Widmer, Daniel Raederstorff

**Affiliations:** DSM Nutritional Products Ltd., Department of Human Nutrition & Health, P.O. Box 2676 Basel, CH-4002 Switzerland

**Keywords:** Chemokines, Cytokines, Endothelial dysfunction, Human umbilical vein endothelial cells, Inflammation, Nutrients, Peripheral blood leukocytes, THP-1 cells, RAW 264.7 cells, Resveratrol

## Abstract

**Background:**

The phenolic substance resveratrol (RES) is a plant metabolite known to modulate numerous physiological functions and to exert beneficial effects as a cancer-chemopreventing agent and on neurological, hepatic, and cardiovascular systems. Since the compound affects the lifespan of yeast and flies it might be an anti-aging substance. Mechanistically, RES is involved in down regulating the inflammatory response. The pleiotropic effects of RES in cells of the immune and endothelial system were examined in this study.

**Results:**

Murine macrophages (RAW264.7 cells), human monocytic/leukemia cells (THP-1), PBLs and HUVECs were incubated with RES and activated with inflammatory stimuli such as LPS or TNF-α. Inflammatory mediators and adhesion molecules were measured by multiplex analysis and gene expression was quantified by RT-PCR. In PBLs, which were activated with LPS, RES blunted the production of TNF-α, CCL2/MCP-1, CCL5/RANTES, CXCL8/IL-8, whereas it increased the production of IL-1β, IL-6, CCL4/MIP-1β and CXCL10/IP-10. Thus, in the blood compartment chemokines attracting mainly monocytes were up-regulated by RES, while those attracting T lymphocytes or neutrophils were diminished. At conditions of endothelial dysfunction (ED), RES reduced the expression of cytokines, chemokines, ICAM and GM-CSF in TNF-α activated HUVECs, whereas eNOS expression was corrected to pre-ED homeostasis. In macrophages nitric oxide, PGE_2_, cytokines (TNF-α, IL-1β, IL-6) and chemokines (CCL2/MCP-1, CCL4/MIP-1β, CCL5/RANTES, CXCL10/IP-10) were reduced by the phenolic substance.

**Conclusions:**

RES had cell-specific and context-dependent effects, in particular on the expression of IL-1β, IL-6, CCL4/MIP-1β and CXCL10/IP-10. It enhanced cellular features that mirror increased alertness to disturbed immune homeostasis in the vascular-endothelial compartment (e.g. increased production of IL-1β or IL-6), whereas it blunted inflammatory mediators in macrophages and consequently chronic inflammation. We infer from the present in vitro study, that RES has unique properties in the regulation of inflammatory and immune responses, which are controlled in a complex hierarchical and temporal order.

**Electronic supplementary material:**

The online version of this article (doi:10.1186/s12906-017-1823-z) contains supplementary material, which is available to authorized users.

## Background

Numerous epidemiological studies have shown that a diet rich in fruits has beneficial effects on diseases such as cardiovascular diseases (CVD), diabetes, obesity and neurodegeneration. Low-grade inflammation is a common feature of these chronic diseases. As a consequence the production of mediators of inflammation is unbalanced and cellular homeostasis is markedly disturbed. These changes influence cell metabolism and alter tissue functions that are supposed to favor disease progression [1]. A hallmark of acute inflammation is the increased production of cytokines and chemokines. These enable and enhance inflammatory processes that support the recruitment of immune cells to the sites of inflammation and eliminate pathogens. Some mediators are further required during the resolution of inflammation [2] or in the differentiation of cells that orchestrate the resolution of inflammatory processes like alternatively activated macrophages [3]. During chronic inflammation a status of un**-**coordinated production of mediators and metabolites is perpetuated and causes tissue and organ damage. Consequently, inflammatory processes have an intrinsic dual nature during acute and chronic inflammation. Given the fact that diet contributes to health homeostasis, nutrients that favorably modulate the inflammatory status are supposed to prevent health disorders and diseases [4].

We investigated the in vitro effects of the phytoalexin resveratrol (RES) in acute and chronic inflammatory responses. In order to cover a potentially wide range of actions in different systemic contexts, we analyzed the effects of RES in three distinct cell types, i.e. human peripheral blood leukocytes (PBLs), human umbilical vein endothelial cells (HUVECs) and murine and human macrophage cells (RAW264.7 cells, THP-1 cells). RES has a broad pattern of biological activities related to metabolic diseases and ageing [5–7]. We show in this study, that RES markedly altered the production of cytokines and chemokines, which regulate the acute inflammatory response in peripheral blood, endothelial cells and macrophages. RES orchestrated the response of cells to inflammatory stimuli in a cell- and compartment-specific way. Notably, RES differentially regulated the expression of inflammatory genes in various compartments of the systemic response to inflammation.

## Methods

### Reagents

Resveratrol (RES), *E. coli* lipopolysaccharide (LPS, serotype 055:B5) and fetal bovine serum (FBS) were from Sigma/Aldrich (Saint-Louis, MO). Cell culture media (RPMI 1640, DMEM), 2-mercaptoethanol and non-essential amino acids (NEAA) were from Invitrogen (Carlsbad, CA). Recombinant human interleukin (IL)-1β, interferon (IFN)-γ and Tumor Necrosis Factor (TNF)-α were from PeproTech EC (London, UK).

### Cell culture

Murine macrophage RAW264.7 and human PBLs have been cultured and treated with inflammatory stimuli as described [8, 9]. Briefly, RAW264.7 cells were seeded into 12-well or 96- well plates at 1 and 0.05 × 10^6^ cells per well, respectively, for 2 days of preculture, starved in DMEM containing 0.25% FBS 18 h before the treatment and stimulated with LPS (1 μg/mL) for 4–24 h in phenol red-free DMEM containing 0.25% FBS [8]. PBLs from healthy donors (8 × 10^6^ viable cells/mL) were cultured in phenol-red free RPMI 1640 (containing 0.25% FBS, 0.1 mM NEAA, 50 U/mL penicillin, 50 μg/mL streptomycin, 50 μM 2-mercaptoethanol) and stimulated with LPS/INF-γ (100 ng/mL, 20 U/mL) with graded amounts of test substances (i.e. series of 1.56 to 50 μM, prepared in two-fold dilution steps from 50 μM solution). RES was added to cultures shortly before starting the incubation. After 2–12 h of culture cells were lysed in RLT buffer (Qiagen, Hilden, Germany) and total RNA was extracted. In order to measure secreted mediators and proteins, cells were cultured for 24 h. Supernatants were then collected and stored at −80 °C until use.

THP-1 cells (from Cell Lines Service Eppelheim, Germany) were maintained at <2 × 10^5^ cells/mL in RPMI 1640 medium supplemented with 50 U/mL penicillin, 50 μg/mL streptomycin, 10% FCS and 2 mM L-glutamine. Cells were treated with 50 nM phorbol 12-myristate 13-acetate (PMA) to induce adherence and differentiation into macrophages. After 2 days of culture, cells were incubated with RES and activated with LPS/IFN-γ (100 ng/mL LPS, 20 U/mL IFN-γ). For gene expression analysis, total RNA was extracted from THP-1 cells 4 h after activation. Cell culture supernatants were recovered after 24 h and processed for chemokine and cytokine analysis.

HUVECs were from Lonza, (Walkersville, MD), cultured in EGM (Endothelial Growth Medium, Lonza) and used for experiments between passages 3 to 7. Cells (2 × 10^5^ per well) were seeded into BioCoat™ Collagen I 6-well plates (Becton Dickinson, San Jose, CA). Cells were activated with TNF-α (10 ng/mL) or IL-1β (5 ng/mL) and cultured for 2–24 h. All treatments were done in duplicate and all experimental series were done at least twice.

### RNA isolation, cDNA synthesis and quantitative RT-PCR

The isolation of total RNA, synthesis of cDNA and quantitative RT-PCR has been performed and quantified as detailed before [9]. Sequences of primers and probes used for Taqman™ gene expression analysis are given in Additional File 1. Results were obtained from triplicates and are indicated as fold changes (± errors, calculated with a logarithmic function) (see [9]).

### Multiparametric analysis of cytokines, chemokines and interleukins

Multiparametric kits for the quantification of chemokines, cytokines and interleukins were purchased from BIO-RAD Laboratories (Hercules, CA) and used in the LiquiChip Workstation IS 200 (Qiagen, Hilden, Germany). Data evaluation was made with the LiquiChip Analyser software (Qiagen) [10]. Nitric oxide (NO), which is quantified as the stable nitrite (NO_2_) by the Griess reaction, and prostaglandin E_2_ (PGE_2_) were measured as described previously [8].

### Statistical analysis

Data were evaluated with the statistical tools described previously [9, 10]. A *p* value <0.05 (calculated by using Student’s t test or one-way ANOVA) was considered to reflect statistically significant differences. Where appropriate, the Tuckey post-hoc test was applied for multiple comparisons. Statistical analyses were performed with SPSS 23.0.0.0 (SPSS, Munich, Germany).

## Results

### Anti-inflammatory effects of RES in murine RAW 264.7 cells

**Fig. 1:**
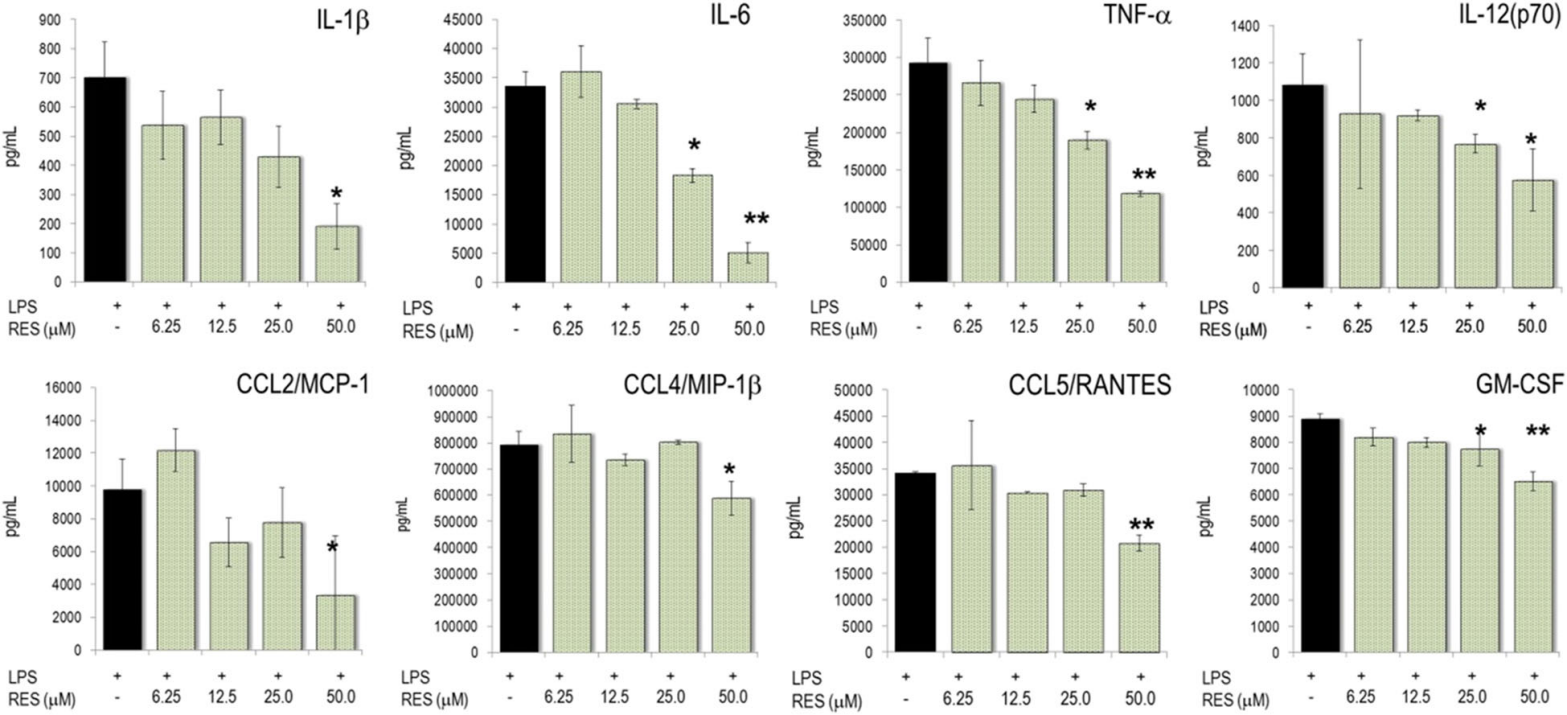
RES modulated cytokine and chemokine production in RAW264.7 cells. Cells were incubated with different concentrations of RES, stimulated with 1 μg/mL LPS and cultured for 24 h. The mediators were determined in cell culture supernatants. Results are shown as mean (± SEM) of triplicate cultures of three experimental series. * *p* < 0.05, ** *p* < 0.01 (versus LPS-stimulated cells)

Initially, we studied the influence of RES on the inflammatory profile of LPS-stimulated murine macrophages (RAW267.4 cells), which express numerous inflammatory genes [11]. RES reduced the production of nitric oxide (NO) and the secretion of COX-2 dependent prostaglandin E_2_ (PGE_2_) (Table 1) (IC_50_ = 27.7 ± 1.6 μM [*n* = 24] and 19.0 ± 2.4 μM [*n* = 20] for NO and PGE_2_, respectively). Furthermore, we analyzed the effects of RES on cytokine and chemokine (CK) secretion in LPS-activated macrophages. RES blunted secretion of IL-1β, IL-6, IL-12(p70), and TNF-α. At 25–50 μM, it also significantly reduced production of CCL5/RANTES, but had only marginal effect on CCL2/MCP-1 and CCL4/MIP-1β (Fig. 1). Notably, RES also impaired LPS-induced GM-CSF production in the murine macrophage cell line.

**Table 1:** Production of PGE_2_ and nitric oxide in LPS-stimulated RAW264.7 cells

*Metabolite*	*LPS alone*	*LPS + RES (25* μM*)*	*p*
PGE_2_ [pg/mL]	6334 ± 306	2816 ± 134	0.010
Nitric oxide (μM)	20.1 ± 0.8	12.1 ± 0.4	0.005

Cells were incubated with 25 μM RES, stimulated with 1 μg/mL LPS and cultured for 24 h. ‘LPS alone’: indicates the value obtained from LPS-stimulated cells (without RES). Results are shown as mean (± SEM) of triplicate cultures obtained in 5 independent experimental series. *p* values were calculated between treatments ‘LPS-alone’ versus ‘LPS + RES’. Unstimulated cells produced metabolites at <10% of LPS-stimulated cells

**Fig. 2:**
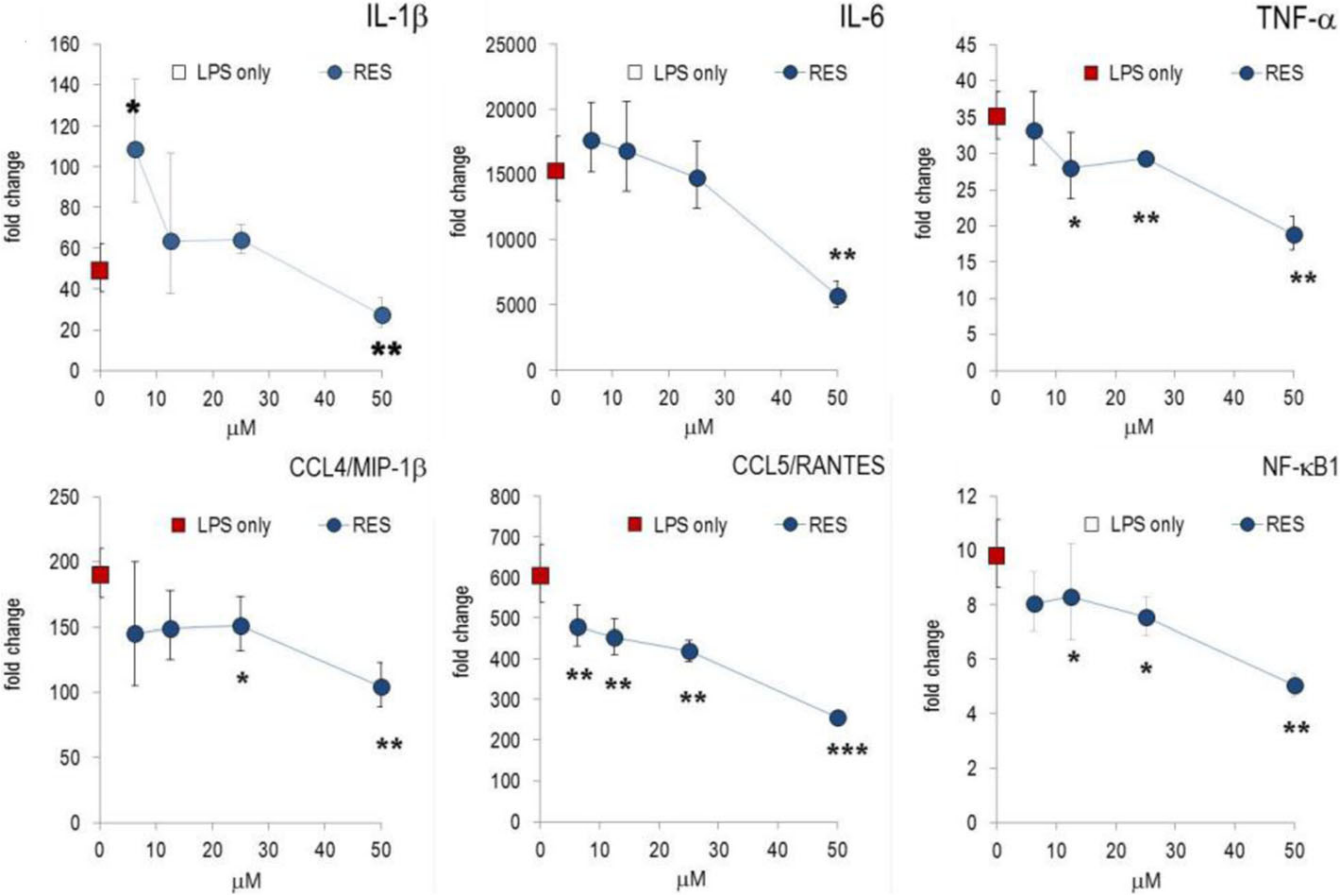
RES modified gene expression in LPS-stimulated RAW264.7 cells. Cells were incubated with RES, and stimulated with 1 μg/mL LPS and cultured for 4 h. Gene expression was quantified by RT-PCR and the data expressed as fold change compared to levels observed in unstimulated cells. Results are shown as mean of triplicates (± errors) (see reference [11]). ‘LPS alone’: indicates the value obtained from LPS-stimulated cells (without substance) and is indicated on the y-axis. * *p* < 0.05, ** *p* < 0.01, *** *p* < 0.001 (versus LPS-stimulated cells)

Next, we investigated whether the expression of inflammatory genes was influenced by RES in RAW264.7 cells. RES diminished mRNA levels of IL-1α, IL-1β, IL-6, TNF-α, CCL4/MIP-1β and CCL5/RANTES (Fig. 2). RES exerted significantly stronger effects at the transcriptional level as compared to protein secretion, since effects were observed at RES concentrations as low as 6.25 μM. RES also impaired the NF-κB transcription pathway, since it reduced the transcription level of NF-κB1 (Table S1). This is consistent with data reported by Tsai et al. [12].

### RES altered the cytokine and chemokine profile in human THP-1 cells

**Fig. 3:**
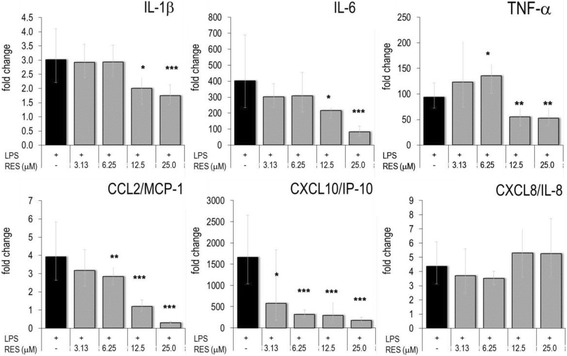
Effect of RES on gene expression in activated THP-1. PMA-treated THP-1 cells were cultured in the presence of indicated concentrations of RES and activated with LPS for 4 h. RT-PCR was performed and the gene expression levels were indicated as mean fold changes (± errors) (versus unstimulated cell) (see also reference [11]). * *p* < 0.05, ** *p* < 0.01, *** *p* < 0.001 (versus LPS-stimulated cells)

Upon treatment with phorbol-myristate acetate (PMA), the human monocytic leukemia cell line THP-1 expressed macrophage features [13]. RES has been found to modulate various biochemical parameters of THP-1 cells [14–16]. In this study we analyzed the impact of RES on inflammatory mediators produced by in vitro differentiated THP-1 cells. When activated with LPS, THP-1 cells up-regulated cytokine and chemokine genes (Fig. 3) and secreted large amounts of PGE_2_, cytokines and chemokines, although not as abundantly as RAW264.7 cells (Table 2). RES was a potent inhibitor of PGE_2_ production in THP-1 cells for which an IC_50_ value of 5.0 ± 1.2 μM (*N* = 5) was calculated. At 25 μM RES almost completely blunted PGE_2_ production (Table 2). Also, IL-1β, IL-6 and CXCL10/IP-10 were significantly decreased by 25 μM RES. In LPS-activated THP-1 cells, RES significantly down-regulated gene expression levels of IL-1β, IL-6, TNF-α and chemokines including CCL2/MCP-1 and CXCL10/IP-10 (Fig. 3). It should be noted that RES selectively altered expression of these chemokines, since others, like CXCL/8/IL-8, were not modified by the polyphenolic compound.

**Table 2:** Resveratrol reduced the secretion of cytokines and chemokines in LPS-activated THP-1 cells

Treatment	PGE_2_	*p*	IL-1β	*p*	IL-6	*p*	TNF-α	*p*	CXCL10/IP-10	
	pg/mL		pg/mL		ng/mL		pg/mL		pg/mL	*p*
LPS alone	3010 ± 333		4225 ± 261		566 ± 9		12,050 ± 70		20,350 ± 353	
LPS + RES (25 μM)	515 ± 65	0.001	2095 ± 21	0.034	75 ± 4	0.013	11,750 ± 495	0.590	14,700 ± 707	0.028

**P**MA-treated THP-1 cells were activated with LPS in the absence or presence of 25 μM RES. The secreted metabolites were measured after 24 h of culture. Mean values ±SD of triplicate cultures are shown. *p* values were calculated between treatments ‘LPS-alone’ versus ‘LPS + RES’

### RES impaired the inflammatory response of LPS-activated PBLs

**Fig. 4:**
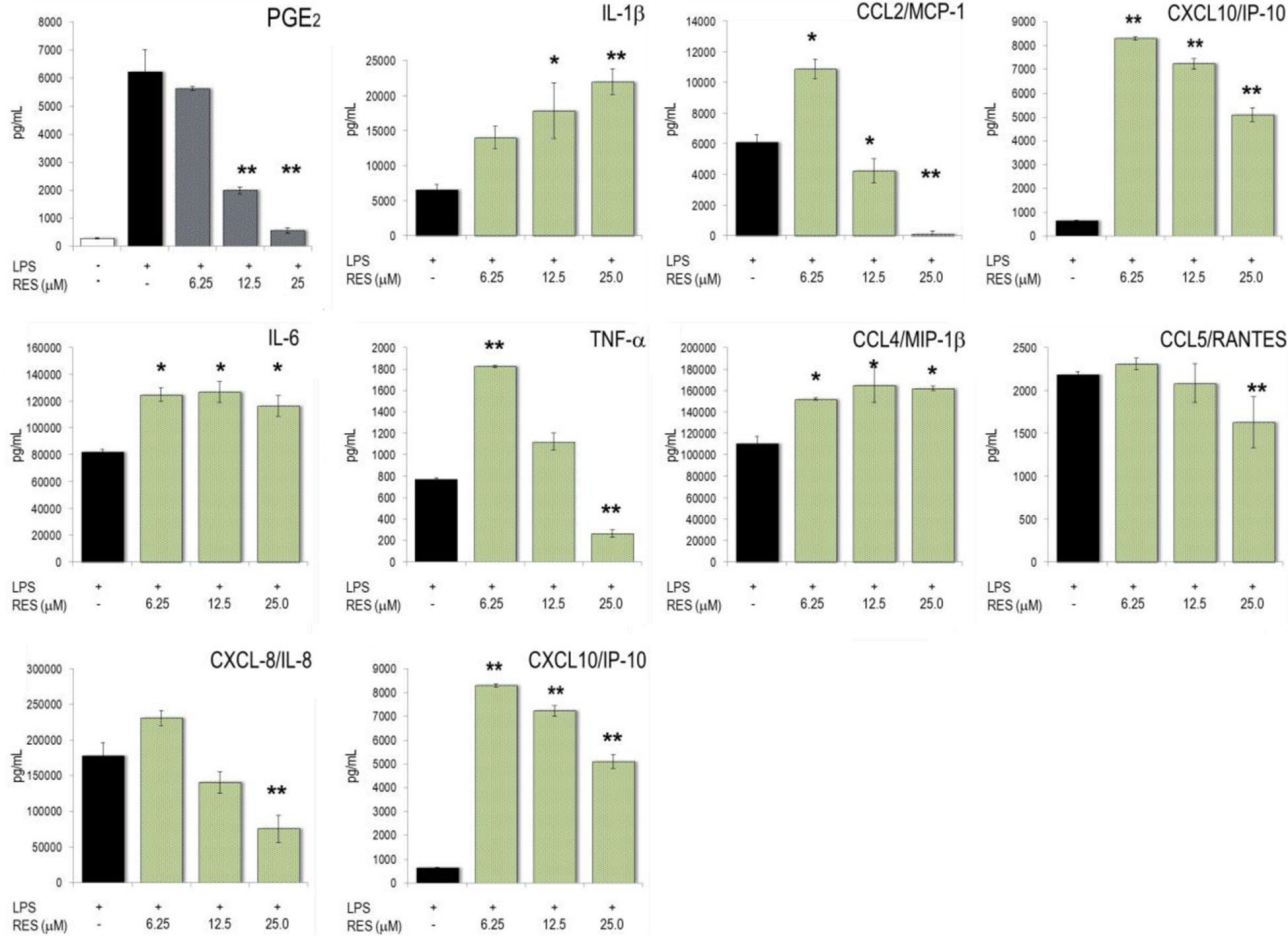
RES altered the production of inflammatory mediators in activated PBLs. Freshly isolated PBLs were incubated with substances, stimulated with LPS and cultured for 24 h. The mediators were determined in cell culture supernatants. Results (± SEM) obtained from cultures of PBLs from three donors are given. *p < 0.05, **p < 0.01 (versus LPS-stimulated cells)

RES is rapidly absorbed from the diet and it reaches peak levels in the blood within 1–6 h after ingestion [17]. This prompted us to more extensively analyze the impact of RES on inflammatory parameters of blood cells. To this aim, human PBLs were stimulated with LPS/INF-γ in the presence of graded amounts of RES and the inflammatory response was quantified by measuring secreted mediators and gene expression. RES concentration-dependently inhibited PGE_2_ production (Fig. 4 and Table 3). RES had equivocal effects on TNF-α secretion, since high concentrations reduced it, whereas low concentrations rather enhanced it.

**Table 3:** RES-induced changes of the chemokine and cytokine profile in human PBLs

*Metabolite*	*LPS/INF-γ alone*	*RES (25* μM*) + LPS/INF-γ*	*p*
PGE_2_ [pg/mL]	4975 ± 483	612 ± 238	0.005
IL-6 [ng/mL]	76.8 ± 5.5	135.1 ± 16.7	0.044
IL-1β [ng/mL]	11.6 ± 1.3	22.3 ± 2.2	0.009
TNF-α [pg/mL]	2762 ± 393	1195 ± 320	0.04
CCL2/MCP-1 [pg/mL]	804 ± 60	352 ± 26	0.002
CCL4/MIP-1β [ng/mL]	70.8 ± 6.2	123 ± 22	0.16
CCL5/RANTES [pg/mL]	1032 ± 220	798 ± 59	0.05
CXCL10/IP-10 [pg/mL]	2072 ± 110	16,758 ± 2649	0.026
GM-CSF	17.9 ± 3.3	15.2 ± 3.0	0.29

Freshly isolated PBLs were stimulated with LPS/INF-γ in the presence of RES and cultured for 24 h. Metabolites were determined in the culture supernatants by multiplex ELISA. Mean (±SD) of triplicate cultures from three donors are shown. *p* values were calculated between treatments ‘LPS/INF-γ -alone’ versus ‘RES + LPS/INF-γ’

With regard to CKs, RES (at >12.5 μM) inhibited CCL2/MCP-1, CCL5/RANTES and CXCL/IL-8 (Fig. 4 and Table 3). On the other hand, RES *stimulated* the secretion of CCL4/MIP-1β and CXCL10/IP-10. The production of IL-1β and IL-6 was concentration-dependently augmented by RES; this is in agreement with previous results [9]. In addition, RES had an overall inhibitory effect on the cytokines and chemokines produced by un-activated PBLs, which secreted only a small fraction of mediators compared to those produced by LPS-stimulated PBLs (Additional file 1: Figure S1). It should be emphasized that cell viability was not impaired at all tested RES concentrations (Additional file 1: Figure S2). PBL monocytes produced detectable amounts of GM-CSF after LPS-activation. Different from the observations made with murine macrophages, RES (at 25 μM) did not impair GM-CSF production in activated PBLs from different donors (Table 3).

**Fig. 5:**
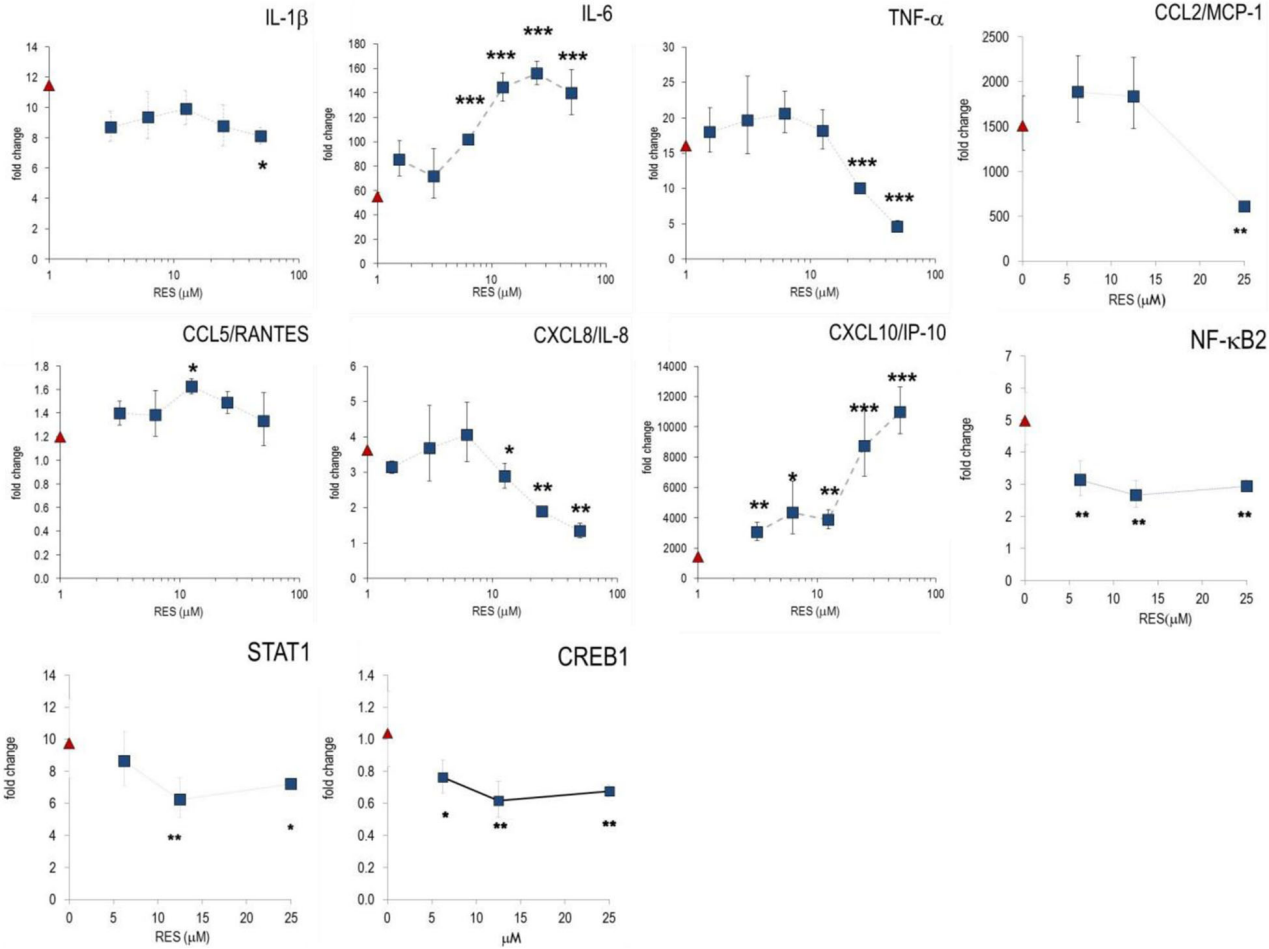
Effect of RES on gene expression in activated PBLs. Freshly isolated PBLs from a healthy donor were incubated with RES and stimulated with LPS for 1-12 h. Gene expression was quantified by RT-PCR and the data expressed as fold change compared to levels observed in unstimulated cells. Mean ± errors (see reference [11]) of triplicate cell culture are given. Only the time point, where maximal stimulation for a given gene was observed, is shown. * *p* < 0.05, ** *p* < 0.01, *** *p* < 0.001 (versus LPS-stimulated cells)

Next, we investigated inflammatory gene expression in human PBLs and studied the impact of RES during the early phase of the inflammatory response. PBLs responded to LPS stimulation by the expression of thousands of genes [11]. Inflammatory genes including IL-1β, IL-6, TNF-α or CXCL8/IL-8 were markedly up-regulated within 2 h of stimulation (Fig. 5) (see also [9]). RES drastically altered the expression levels of cytokines and chemokine genes, since TNF-α and CXCL8/IL-8 were significantly reduced. Conversely, it strongly up-regulated e.g. IL-6 and CXCL10/IP-10. IL-1β expression was not modified by RES. CCL2/MCP-1 mRNA levels were lower in 25 μM RES-treated LPS-activated PBL. It should be emphasized that CCL5/RANTES gene expression was not induced by LPS, nor was it affected by RES (Fig. 5).

Many of LPS-induced genes in PBLs are regulated by transcription factors (TF). Accordingly, LPS up-regulated TFs of the NF-κB pathway and STAT. When PBLs were activated in the presence of RES, mRNA levels of NF-κB2, STAT1 and CREB1 were reduced (Fig. 5). Collectively, the data indicate that RES modulated cytokine and chemokine expression at the transcriptional level via TFs.

### Modulation of inflammatory parameters associated with endothelial dysfunction

**Fig. 6:**
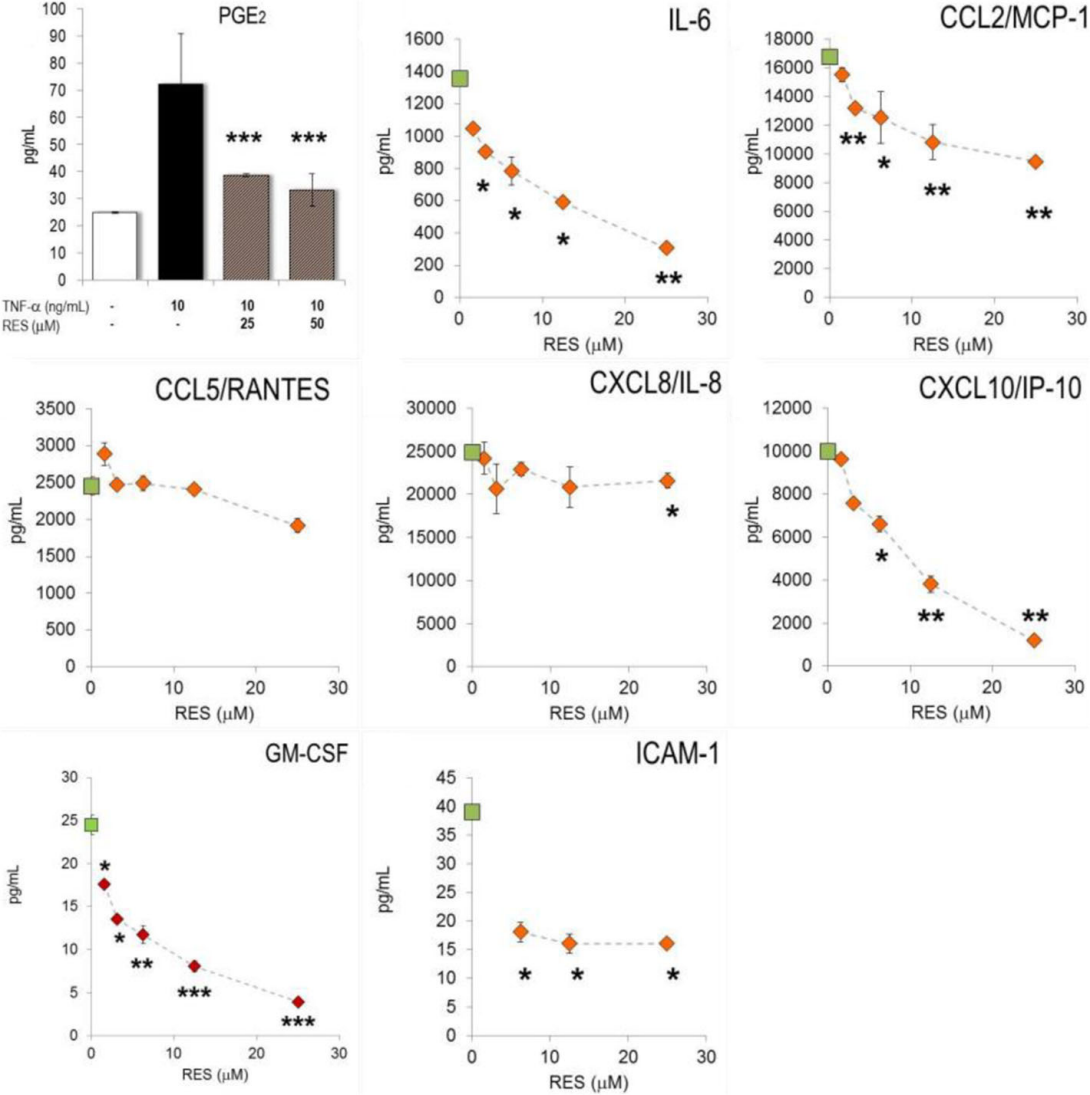
Secretion of mediators by activated HUVECs. Cells were incubated with RES, activated with 10 ng/mL TNF-α and cultured for 24 h. The secreted cytokines, chemokines, ICAM-1 and VCAM-1 were quantified by Luminex technology (see Methods). Mean ± SD of triplicates are given. Unstimulated cells produced <10% of the mediators secreted by TNF-α stimulated cells. * *p* < 0.05, ** *p* < 0.01 *** *p* < 0.001 (versus TNF-α -stimulated cells)

Several studies previously showed that RES modified biological features of the vascular-endothelial compartment (see e.g. [18]). Therefore, we examined the effects of RES on inflammatory features of the endothelial cells. To this aim, we induced endothelial dysfunction (ED) by activating HUVECs with TNF-α or IL-1β and measured the impact of RES in these cells (Fig. 6). Stimulation of HUVECs with TNF-α induced an up to ~100-fold increase of inflammatory mediators (see Legend to Fig. 6). RES significantly diminished the secretion of PGE_2_, IL-6, CCL2/MCP-1, and CXCL10/IP-10, whereas CCL5/RANTES and CXCL8/IL-8 were resilient to RES treatment. Pro-inflammatory stimuli (i.e. TNF-α) also induced the production of GM-CSF in HUVECs, where it promoted the differentiation of macrophages into foam cells [19]. GM-CSF production by TNF-α activated HUVECs was virtually abrogated by high concentrations of RES (Fig. 6). Unstimulated HUVECs secreted low amounts of metabolites (Additional file 1: Table S2).

**Fig. 7:**
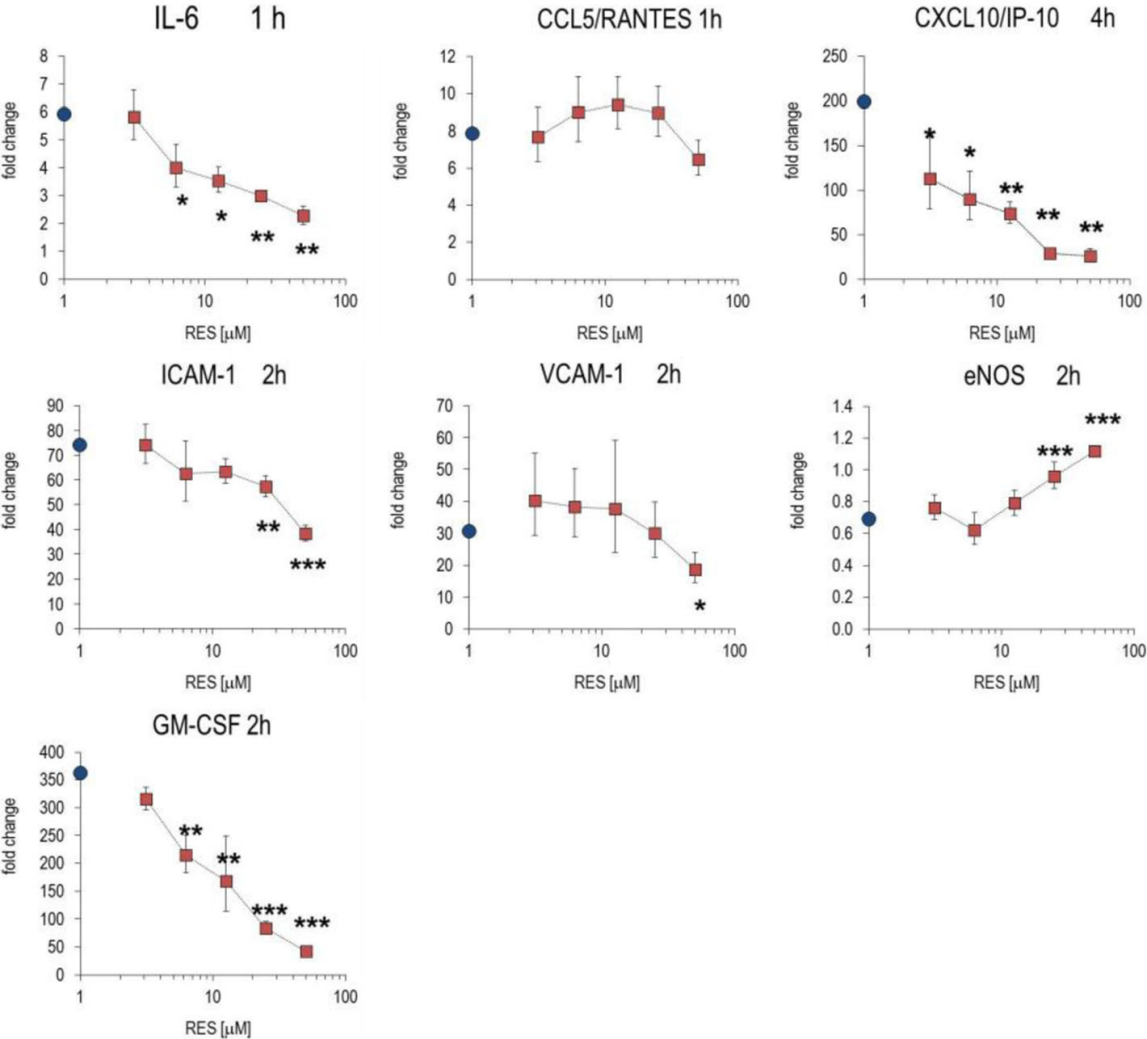
Impact of RES on gene expression in activated HUVECs. Cells were incubated with substances, activated with 10 ng/mL TNF-α and cultured for 1, 2 and 4 h. Gene expression was quantified by RT-PCR and the data expressed as fold change compared to levels observed in unstimulated cells (set as 1). The data at the time point of maximal changes in gene expression are shown (indicated in the panels). Mean ± errors (see ref. [11]) of triplicate cell cultures are given. * *p* < 0.05, ** *p* < 0.01 *** *p* < 0.001 (versus TNF-α -stimulated cells). Symbol on the y-axis show the values obtained with ‘TNF-α alone’ treated HUVECs

TNF-α activation of HUVECs induced marked up-regulation of gene expression of IL-6, ICAM-1, VCAM-1, CCL2/MCP-1, CXCL8/IL-8 and CXCL10/IP-10 (Fig. 7). Most of these genes were concentration-dependently regulated by RES, which significantly decreased expression levels of IL-6 and CXCL10/IP-10. Unlike VCAM-1 expression, ICAM-1 gene expression was significantly reduced by RES. Moreover, RES reduced GM-CSF gene expression to levels observed with unstimulated cells. This is consistent with the features obtained at the level of secretion of inflammatory mediators. Another characteristic of ED induced by TNF-α is the down-regulation of endothelial nitric oxide synthase (eNOS) in HUVECs. Accordingly, TNF-α treatment diminished eNOS gene expression, while RES moved eNOS expression back to homeostatic levels (see also [20]).

## Discussion

**Fig. 8:**
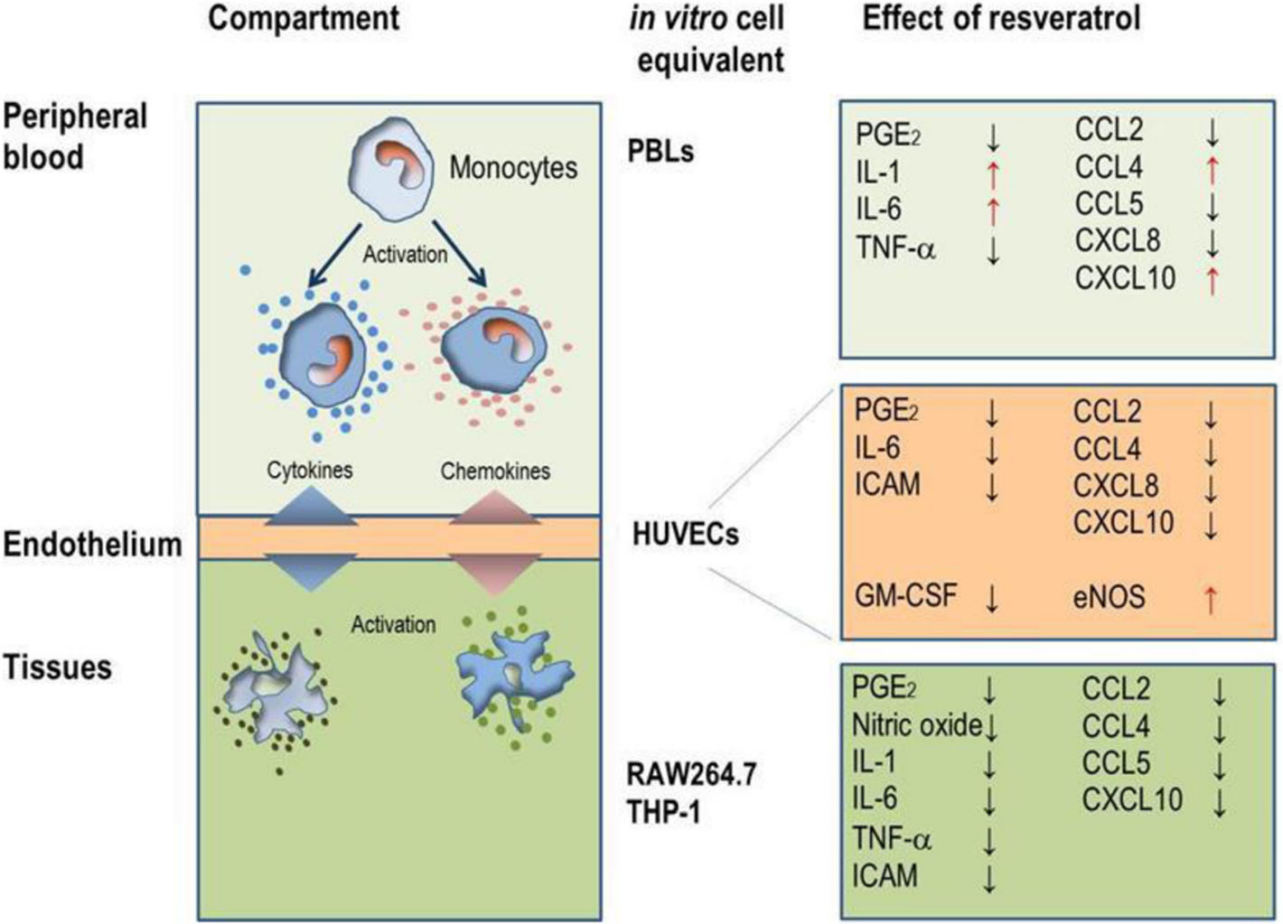
Context-dependent and cell-specific effects of RES. Peripheral blood monocytes respond to inflammatory stimuli by increased production of cytokines and chemokines. Similarly, endothelial cells secrete large amounts of inflammatory metabolites at conditions of endothelial dysfunction induced by TNF-α. Activated monocytes trans-migrate through endothelial layers and differentiate into tissue-resident macrophages. During acute or chronic inflammation, macrophages respond to stimuli by the production or release of numerous cytokines and chemokines. RES blunts (*black arrows*) or enhances (*red arrows*) cytokines, chemokines and adhesion molecules and thus intervenes in trafficking, activation and differentiation of immune cells in different compartments

The biological activities of RES have been evaluated in a plethora of studies (for recent reviews see [5–7, 21]). Hence, RES becomes one of the most intensely examined plant secondary metabolite ever. Not unexpectedly, many controversies have grown [6], since the effects of RES were pleiotropic and relied on diverse mode of actions in different cells, tissue, metabolic states and species [21]. In this study we compared the effects of RES on the production of chemokines and cytokines in different cell types, which represent the innate immune system (i.e. macrophages) and the vascular-endothelial compartment (i.e. PBLs and HUVECs, respectively). The observed data require a revisited holistic view of the polyphenolic substance, in which the various features of RES are connected with their temporal and systemic occurrence in the inflammatory and immune response (Fig. 8).

During the body’s response to homeostatic changes due to external and internal causes, a large cascade of metabolic and cellular events occurs and reveals the status of inflammation. The subsequent cell migration during the innate and adaptive immune response is orchestrated and controlled by cytokines and CKs. CCL2/MCP-1, CCL3/MIP-1α and CCL4/MIP-1β recruit monocytes. CCL5/RANTES and CXCL10/IP-10 enable chemotaxis of activated T lymphocytes. Neutrophils are recruited by CXCL8/IL-8 [22]. A major and novel feature of the present study relies on the observation that RES cell-specifically modulated the chemokine production; in the blood compartment (i.e. PBLs), RES dampened the production of CCL5/RANTES and CXCL8/IL-8, but enhanced CCL4/MIP-1β and CXCL10/IP-10 (Fig. 4). Subpopulations of PBLs such as lymphocytes, monocytes/macrophages and neutrophils responded to LPS at various degrees [9]. Presumably, RES has genuine effects on each of these populations. By altering the delicate balance between CKs, RES might influence the temporal pattern of cell migration during the different phases of the innate immune response. Surprisingly, RES was found to enhance the expression of IL-1β and IL-6 in the peripheral compartment but not in macrophages or in the endothelium (see below).

Upon TNF-α stimulation HUVECs expressed a pattern of cytokines and chemokines, which had many common features with that of LPS-activated PBL. In endothelial cells, RES corrected CK expression in a comparable way as in PBL, although the extent of changes was less striking. RES also reduced the expression of ICAM-1 towards pre-ED homeostasis, whereas VCAM-1 expression was insignificantly influenced by the polyphenol. Thus, a diminished endothelial diapedesis might be one biological consequence of the presence of RES in the blood. Our data are in agreement with other studies, which showed that RES down-regulated ICAM-1 and mitigated chemotaxis and vascular inflammation via CCL2/MCP-1 and NF-κB, respectively [23]. The pleiotropic effects of RES in HUVECs were further underscored by the RES-dependent increase of eNOS expression at ED conditions [20, 23, 24]. Notably, the production of GM-CSF, a key marker of atheroma formation [19, 25] and for the differentiation of pro-inflammatory macrophages [26], was robustly blunted by the presence of RES. Collectively, the plant metabolite was shown to improve endothelial functions at various levels. Chronic in vivo exposure of dys-regulated endothelium to RES is expected to beneficially modulate vascular function.

Macrophages, which develop from monocytes, are pivotal during initiation and resolution of inflammation and they differentiate into tissue-specific subsets [27]. Nitric oxide (NO) and PGE_2_ are hallmarks of both acute and chronic inflammation in pro-inflammatory macrophages. RES impaired iNOS-dependent NO production in RAW264.7 macrophages (Fig. 1 and [12] and exerted strong inhibitory effects on PGE_2_ and thus clearly met the criteria of an anti-inflammatory nutrient. Likewise, the production and expression of cytokines including TNF-α and the pro-inflammatory IL-1β and IL-6 were impaired by RES, suggesting that it dampened the classical activation of macrophages. With regard to CKs, RES blunted CCL4/MIP-1β and CCL5/RANTES and therefore the recruitment of monocyte and activated T lymphocytes, respectively. Notably GM-CSF, a growth and differentiation factor for pro-inflammatory macrophages, was robustly diminished by RES. These effects were not species-specific, since analogous results were observed with the human monocytic leukemia cell line THP-1. RES altered cell morphology, gene expression, ligand-receptor interactions, signaling pathways and eventually foam-cell formation [14–16, 28]. Collectively, RES had an overall anti-inflammatory profile in macrophages. This is consistent with observations in chondrocytes at conditions that mimic chronic inflammation associated with osteoarthritis [33].

The molecular regulation of inflammatory processes is critically governed by transcription factors (TF). LPS-activation of monocytes and macrophages induced the NF-κB dependent transcription of chemokines such as CXCL8/IL-8, CXCL10/IP-10, CCL2/MCP1 and CCL5/RANTES [29–31]. As shown in this study, RES influenced the transcription level of NF-κB elements as well as STAT1 and CREB1 (for reviews see [5, 32]). Likewise, TNF-α induced activation of the NF-κB elements is modulated by RES and its related substance piceatannol. Further investigations should evaluate whether RES has distinct effects on polymorphonuclear cells, including neutrophils, monocytes/macrophages or lymphocytes.

The idiosyncratic effects of RES in three interconnected cellular systems are summarized in Fig. 8. Except for the peripheral blood compartment, cytokines and chemokine markers are consistently altered in the same direction thus corroborating the favorable properties of RES on attenuating chronic or low-grade inflammation. Notably, PGE_2_ was strongly and consistently blunted in PBLs, macrophages endothelial cells and chondrocytes [9, 33]. Given the widespread importance of PGE_2_ in the regulation of the immune response [34], RES appears to predominantly modulate the immune response by influencing cellular PGE_2_ levels. It should be noted that RES did not significantly change COX-2 gene expression in various cellular systems [9, 33] and hence does not impair the COX-2 dependent production of mediators required during the resolution of inflammation [35]. The cell-specific effect on interleukin production is another salient feature of RES, since it favored production of IL-1β and IL-6 in PBLs, but it had opposite effects in macrophages. Enhanced production of IL-1β and IL-6 is considered to characterize a pro-inflammatory status, but it is also involved in tissue regeneration [36]. In the immune response, however, IL-1β and IL-6 contribute to T_h_ lymphocyte differentiation and function [37, 38]. Consequently, immune cells exposed to RES in the vascular compartment would produce higher levels of IL-1β or IL-6 and thus be primed for the adaptive immune response. Since RES has a short half-life in the blood [39], it exerts peripheral effects on immune cells only for a limited time. The RES-induced enhancement of immune responsiveness by IL-1β or IL-6 is high in blood cells, whereas it is reverted in macrophages, which are key players in low-grade and chronic inflammation. Hence RES contributes to improve the systemic response to ‘danger’ signals [3, 40] in the periphery and concomitantly reduce the low-grade inflammatory status related to chronic diseases in tissues. This would account for genuine and even opposite effects of RES in different biological contexts.

The diverse features of RES demonstrated in cellular systems need to be further evaluated in appropriate nutritional intervention studies. Bakker et al. supplemented overweight subjects for 5 weeks with an ‘anti-inflammatory diet mixture’ (AIM) comprising RES and observed subtle changes in expression levels of IL-12, ICAM-1 and VCAM-1, whereas AIM did not alter in vivo levels of CCL2/MCP-1, CCL3/MIP-1α, CCL5/RANTES, CXCL8/IL-8 or PGE_2_ [4]. It is likely that nutrient-induced changes were no longer sensed during long supplementation periods and thus not measurable. The experimental set-up of the cellular study reflects features of short-term supplementation and thus acute inflammation. As a consequence the effects of nutrients on plasma metabolites or leukocyte gene expression will only be detected in vivo at conditions of immediately perturbed homeostasis as it is observed during food intake. In general, the observed effects required rather high RES concentrations, which are not met in plasma or only for a short period of time after food intake. Yet, it should be noted that in cellular and tissue compartments RES reached substantially larger levels [41].

## Conclusions

This study shows that resveratrol modulates many mediators of the inflammatory response. Its effects are context-dependent, i.e. RES might influence chemokines and cytokines in opposite ways in different tissues. While we corroborate the overall anti-inflammatory effect of the polyphenol, we nuance it along a biological divide where beneficial effects might also imply an enhancement of a pro-inflammatory property which is required to rapidly respond to, and resolve, an acute inflammation.

## Additional file

Additional file 1: Supplementary Information. (DOCX 117 kb)

## Abbreviations

CCL: CC chemokine ligand
CK: chemokines
CXCL: CXC chemokine ligand
GM-CSF: Granulocyte-macrophage colony-stimulating factor
HUVECs: Human umbilical vein endothelial cells
IL: Interleukin
LPS: Lipopolysaccharide
NO: Nitric oxide
PBLs: Peripheral blood leukocytes
PGE_2_: Prostaglandin E_2_
RES: Resveratrol
