# Supplementary material for: Resveratrol distinctively modulates the inflammatory profiles of immune and endothelial cells
Source: BMC Complement Altern Med. 2017 Jun 13;17:309. doi: 10.1186/s12906-017-1823-z (PMC5470273; doi:10.1186/s12906-017-1823-z)
Supplement: Additional file 1: — Supplementary Information. (DOCX 117 kb) [file 12906_2017_1823_MOESM1_ESM.docx]

Supplementary Information

**T**able S1: RES altered the expression of inflammatory genes in RAW264.7 cells

RAW264.7 cells were stimulated with 1 μg/mL LPS in the presence of the indicated substances and cultured for 4h. Gene expression was quantified by RT-PCR. Mean values ± SD (of triplicate) are given.

| *Gene* | *Fold change*  *LPS stimulated* | *Fold change*  *RES (25 μM) + LPS-stimulated* | *p value* |
| --- | --- | --- | --- |
| IL-1α | 23116 ± 3620 | 14096 ± 1569 | 0.002 |
| TNF-α | 35.2 ± 3.4 | 29.4 ± 0.9 | 0.049 |
| CCL4/MIP-1β | 191 ± 21 | 151 ± 17 | 0.030 |
| CCL5/RANTES | 606 ± 75 | 452 ± 45 | 0.001 |
| NF-κB1 | 9.8 ± 1.3 | 7.5 ± 0.7 | 0.021 |

**T**able S2: Effects of resveratrol on secretion of inflammatory metabolites of unstimulated HUVECs

Human umbilical vein endothelial cells were cultured in the presence of graded amounts of RES 24h. The amount of metabolites in the culture supernatant was determined by EIA and multiplex ELISA. Mean values ± SD (of triplicate cultures) are given.

| *Metabolite* | *Unstimulated* | *Unstimulated + RES (25 μM)* |
| --- | --- | --- |
|  |  |  |
| PGE_2_ | 55 ± 29 ^1)^ | 86±15 |
| IL-6 | 30 ± 1 | 7±0 |
| CCL2/MCP-1 | 291 ± 57 | 159±26 |
| CCL5/RANTES | 3 ± 1 | 3±0 |
| CXCL8/IL-8 | 1345 ± 148 | 606±29 |
| CXCL10/IP-10 | 1 ± 0 | <LOD ^2)^ |

^1)^ pg / mL, ^2)^ limit of detection

**F**igure S1: Production of PGE_2_, cytokines and chemokines by unstimulated PBL

**F**reshly isolated PBLs were cultured for 24h without or with the 25 μM RES. Secreted metabolites were determined by multiplex analysis. Mean-values (± SD) of triplicate cultures are given. Similar observations were made with PBLs obtained from three different donors.


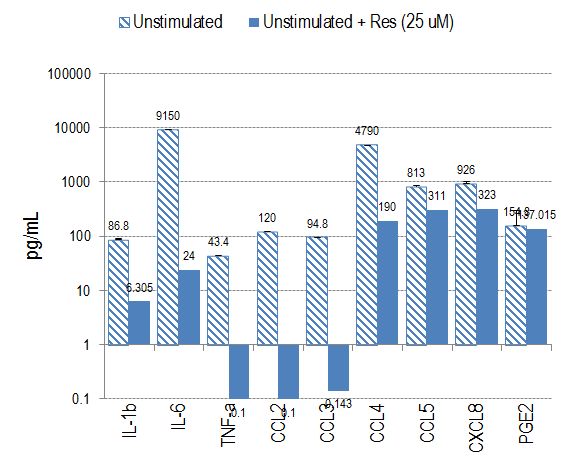


**F**igure S2: Viability of PBL is not affected by RES

**F**reshly isolated PBLs were incubated with the indicated amounts of RES for 24 h. LDH released into the culture medium was measured with the commercially available assay system (Promega) (upper panel) and is expressed as % of total cellular LDH. Alternatively, the Alamar Blue® cell viability test was applied (lower panel) following the instructions of the manufacturer (ThermoFischer Scientific).

# Synopsis of primers and probes used for RT-PCR in this study

human genes

| *Gene* | *Forward primer sequence* | *Reverse primer sequence* | *Probe sequence* |
| --- | --- | --- | --- |
| IL-1beta | ggcctcaaggaaaagaatctgtac | gggatctacactctccagctgtaga | tcctgcgtgttgaaagatgataagccca |
| IL-6 | TGACAAACAAATTCGGTACATCCT | TCTGCCAGTGCCTCTTTGCT | CAGCCCTGAGAAAGGAGACATGTAACAA |
| TNF-alpha | TCGAACCCCGAGTGACAA | AGCTGCCCCTCAGCTTG | CCTGTAGCCCATGTTGTAGCAAACCC |
| CCL2/MCP-1 | TCGCGAGCTATAGAAGAATCACC | CCTTGGCCACAATGGTCTTG | CAGCAAGTGTCCCAAAGAAGCTGTGATCT |
| CCL5/RANTES | ACCAGTGGCAAGTGCTCCA | GCACACACTTGGCGGTTCTT | CCCAGCAGTCGTCTTTGTCACCCG |
| CXCL8/IL-8 | ACTGACATCTAAGTTCTTTAGCACTCC | GCCTTCCTGATTTCTGCAGC | TGGCAAAACTGCACCTTCACACAG |
| CXCL10/IP-10 | TGAAATTATTCCTGCAAGCCAA | CAGACATCTCTTCTCACCCTTCTTT | TGTCCACGTGTTGAGATCATTGCTACAATG |
| GM-CSF | TGATGGCCAGCCACTACAAG | CAAAGGTGATAATCTGGGTTGCA | ACTGCCCTCCAACCCCGGAAACTT |
| eNOS | TGAGTCAGGCCCGGGAC | CGTGGGCCTGGGAGC | TCATCAACCAGTACTACAGCTCCATTAAGAGGAGC |
| VCAM-1 | CAACCGTCTTGGTCAGCCCTTCCTC | GTCATATTCACAGAACTGCCTTCCT | CAACCGTCTTGGTCAGCCCTTCCTC |
| ICAM-1 | GGAGGTCACCCGCAAGGTT | GGCTGCTACCACAGTGATGATG | TGAATGTGCTCTCCCCCCGGTATG |
| STAT1 | CTGAGTTGATTTCTGTGTCTGAAGTTC | CAAACTCCTCAGGAGACATGGG | CCCTTCTAGACTTCAGACCACAGACAACCTGC |

murine genes

| *Gene* | *Forward* | *Reverse* | *Probe* | |
| --- | --- | --- | --- | --- |
| COX-2 | AACATGGACTCACTCAGTTTGTTGA | CCACTGCTTGTACAGCAATTGG | CAGATTGCTGGCCGGGTTGCTGGGGGA |  |
| VCAM | AAAGAACTACAAGTCTACATCTCTCCCAG | CACAGCACCACCCTCTTGAA | CAACGATCTCTGTACATCCCTCCACAAGG |  |
| NF-kB1 | tcgccaggcgacacg | agcaacatcttcacatccccc | caacgcccttttcgactacgcagtg |  |
| NF-kBp49/100 | acctcggcgtcatcaacct | cccagtgattaccgccagg | caaccacctgcaccagacgcctct |  |
| IL-1alpha | tctcagattcacaactgttcgtg | ctggcaactccttcagcaa | cgctcaaggagaagaccagcccgt |  |
| IL-1beta | tgtaatgaaagacggcacacc | ttctttgggtattgcttggga | tccacactctccagctgcaggg |  |
| IL6 | ccagaaaccgctatgaagttcc | caccagcatcagtcccaaga | tctgcaagagacttccatccagttgcc |  |
| IL-12p35 | CAGTCCCGAAACCTGCTG | AGTGCAGGAATAATGTTTCAGTTTT | CTCTGGCCGTCTTCACCATGTCATCTGT |  |
| TNF-alpha | atggcccagaccctcaca | ttgctacgacgtgggctaca | tcagatcatcttctcaaaattcgagtgacaagc |  |
| iNOS | cagctgggctgtacaaacctt | gaatgtgatgtttgcttcggac | cgggcagcctgtgagacctttga |  |
| CCL4/MIP-1beta | ctcttgctcgtggctgcc | gggagggtcagagccca | tctgtgctccagggttctcagcacc |  |
| CCL5/RANTES | GCAAGTGCTCCAATCTTGCA | CTTCTCTGGGTTGGCACACA | CGTGTTTGTCACTCGAAGGAACCGC |  |
| CXCL10/IP-10 | GCCGTCATTTTCTGCCTCA | CGTCCTTGCGAGAGGGATC | CCTGCTGGGTCTGAGTGGGACTCAA |  |

Other genes were measured by low density array (LDA) RT-PCR, for which the primers and probes were not disclosed by the manufacturers (Qiagen).
